# The Association Between Single and Two-Month Average Pre-dialysis Blood Pressure and Left Ventricular Hypertrophy in Patients Undergoing Chronic Hemodialysis: A Prospective Observational Study

**DOI:** 10.7759/cureus.107282

**Published:** 2026-04-18

**Authors:** Adnan Musanovic, Anis Cerovac, Senaid Trnacevic, Demir Bedak

**Affiliations:** 1 Department of Emergency Medicine, Clinical Center University of Sarajevo, Sarajevo, BIH; 2 Department of Obstetric and Gynecology, General Hospital Tešanj, Tešanj, BIH; 3 Internal Medicine, Medical Faculty, University of Tuzla, Tuzla, BIH; 4 Department of Pulmonology, General Hospital Tešanj, Tešanj, BIH

**Keywords:** blood pressure (bp), end stage renal disease (esrd), hemodialys, left ventricular hypertrophy (lvh), trans thoracic echocardiography

## Abstract

Left ventricular hypertrophy (LVH) is common in patients with end-stage renal disease (ESRD) receiving chronic hemodialysis (HD) and is associated with adverse cardiovascular outcomes. Pre-dialysis blood pressure (BP) is routinely measured, but the association of a single in-unit BP compared with longer-term averages remains unclear. In this prospective observational study conducted at the Dialysis Center in Doboj (June-September 2017), 97 chronic HD patients (≥5 months on HD) underwent standardized pre-dialysis BP measurements three times weekly over two months and transthoracic echocardiography. Single BP was defined as the mid-week pre-dialysis measurement, while the two-month average BP represented the mean of all pre-dialysis measurements. Volume overload was estimated clinically from pre-dialysis weight relative to dry weight and averaged over two months. Associations between BP metrics and left ventricular mass index (LVMI) were assessed using linear and multivariable regression adjusted for age, hemoglobin, and two-month average volume overload. Receiver operating characteristic (ROC) analysis evaluated the two-month average systolic BP (SBP) for LVH discrimination.

Among the participants (mean age: 58 ± 12 years), LVH was present in 44/97 (45%). Single pre-dialysis SBP and all diastolic BP (DBP) measures were not significantly associated with LVMI. In contrast, two-month average pre-dialysis SBP was independently associated with LVMI (β = 0.3847, p = 0.0433). A two-month average SBP cutoff of > 150 mmHg discriminated LVH (area under the curve (AUC) 0.619, 95% confidence interval (CI): 0.515-0.717; p = 0.039), with 70.45% sensitivity and 55.77% specificity; SBP > 150 mmHg was associated with higher odds of LVH (odds ratio (OR): 3.11, 95% CI: 1.34-7.24). These findings support longitudinal assessment of pre-dialysis SBP, which was associated with higher LVMI/LVH in this cohort.

## Introduction

Left ventricular hypertrophy (LVH) is a common cardiovascular complication in patients with end-stage renal disease (ESRD) undergoing chronic hemodialysis (HD) and is a strong predictor of morbidity and mortality in this population. It is estimated that up to 75% of patients on maintenance HD exhibit some form of LVH, primarily due to chronic pressure and volume overload, as well as structural and functional vascular abnormalities associated with uremia and dialysis-related hemodynamic shifts [1]. Pre-dialysis blood pressure (BP), measured just before dialysis sessions, is especially critical. Several cohort studies, including the large Predictors of Arrhythmic and Cardiovascular Risk in End-Stage Renal Disease (PACE) Study, have demonstrated that higher pre-dialysis systolic BP (SBP) and diastolic BP (DBP) are significantly and independently associated with increased left ventricular mass index (LVMI).

Specifically, each 10 mmHg rise in SBP corresponds to approximately a 5-7 g/m² increase in LVMI after multivariable adjustment. Notably, these associations persist even after adjusting for volume status and arterial stiffness, indicating a direct pressure load effect on cardiac remodeling [2,3]. Despite the recognition of BP as a key modifiable risk factor and the known benefits of sodium restriction, ultrafiltration, and antihypertensive therapy, guidelines often emphasize volume control over specific pre-dialysis BP targets [4,5,6]. Moreover, while ambulatory BP monitoring and home BP readings are gaining traction as better predictors of cardiovascular events, pre-dialysis SBP remains clinically pivotal and easily accessible [7].

Few well-controlled studies have specifically examined pre-dialysis BP as an independent predictor of LVMI, independent of confounding factors such as volume overload, inflammation, and other hemodynamic variables. Additionally, recent artificial intelligence-based models have identified pre-dialysis SBP as one of the strongest predictors of intradialytic hypotension and hypertension [3,8]. Yet, its relationship with chronic structural cardiac changes remains insufficiently defined. Moreover, the literature on the association between pre-dialysis hypertension and target organ damage is inconsistent, with several studies failing to demonstrate a significant link between pre-dialysis hypertension and left ventricular damage, a key manifestation of target organ injury [9,10]. Despite decades of research into the relationship between BP and outcomes in HD, certain fundamental questions remain unresolved.

However, despite the clinical importance of BP control in HD patients, it remains unclear whether a single in-center pre-dialysis BP measurement adequately reflects the association between BP burden and LVH. In light of this, the present study aimed to assess the association between single versus two-month average pre-dialysis systolic and diastolic BP with LVMI and LVH in patients undergoing chronic HD, and to explore whether an approximate BP threshold emerges within this cohort.

## Materials and methods

Patients

A prospective observational study was conducted among patients enrolled in the chronic HD program at the Dialysis Center in Doboj between June and September 2017. Inclusion criteria were patients receiving HD for at least five months, with a minimum of three months to allow clinical stabilization after entering the chronic dialysis program, and an additional two months for average BP monitoring. Exclusion criteria were as follows: presence of atrial fibrillation (due to unreliable BP measurements), use of a central venous catheter for dialysis, liver failure, uncontrolled diabetes mellitus, pregnancy, concurrent medications affecting the autonomic nervous system, secondary hypertension, and incomplete medical data.

Of the initial 123 patients enrolled, 97 met the inclusion criteria and were included in the final analysis: 34 women (35.1%) and 63 men (64.9%), with a mean age of 58 ± 12 years. A total of 26 patients were excluded during the study: five due to a dialysis duration of less than five months, nine due to permanent atrial fibrillation, two due to the use of a central venous catheter, one due to both atrial fibrillation and catheter use, and nine due to incomplete medical data. For each participant, basic demographic information was collected, a comprehensive medical history was obtained, medical records were reviewed, and a detailed clinical examination was performed.

Blood pressure measurement

BP values were measured using the standard Korotkoff auscultatory method, using a standard aneroid sphygmomanometer. BP was measured before each dialysis session three times weekly over two months. BP measurements were performed by the same trained dialysis personnel according to a standardized protocol in order to reduce inter-observer variability. The single BP refers to the mid-week pre-dialysis measurement, while the two-month average BP represents the average of all pre-dialysis measurements over the entire two-month period. Participants spent five minutes in a seated position before the measurement, with the arm supported at heart level. BP values are expressed in mmHg. The reported BP values were compared with LVMI and analyzed for their association with LVH.

Volume overload assessment

Volume overload was assessed clinically using daily pre-dialysis body weight in relation to the prescribed dry weight, supported by routine clinical examination findings (e.g., peripheral edema, lung auscultation). Volume overload was calculated in relation to the prescribed dry weight, which was periodically reassessed during the observation period according to routine clinical evaluation. The two-month average volume overload was entered into regression analyses as a continuous variable.

Volume overload was expressed as a percentage of dry weight:

$\text{Volume overload (\%)} = \left( \frac{\text{pre-dialysis weight} - \text{dry weight}}{\text{dry weight}} \right) \times 100$

The two-month average volume overload (%) was calculated as the mean of all available pre-dialysis assessments during the two months.

Echocardiography

Echocardiography was performed twice during the study period within a standardized post-dialysis time window, approximately one hour after the dialysis session, and values were averaged for analysis. A General Electric LOGIQ C5 Premium ultrasound machine (General Electric, Boston, MA) equipped with an M3S 1.5-4 MHz phased array transducer was used. The assessments included B-mode imaging, colour Doppler, and pulsed Doppler techniques. Participants were positioned in the left lateral decubitus position to optimize image quality. Standard echocardiographic views were obtained, including parasternal long-axis, parasternal short-axis, apical four-chamber, and five-chamber views. The patients were clinically and biochemically stable in their HD programs and had normal sinus rhythms on their electrocardiograms.

Left ventricular mass was calculated using the Devereux-modified American Society of Echocardiography (ASE) formula from linear measurements (IVSd, LVIDd, and LVPWd). LVMI was obtained by indexing LVM to body surface area. LVH was defined as LVMI > 95 g/m² (women) and > 115 g/m² (men) [11]. All measurements were performed by a certified cardiologist and analyzed using EchoPac PC software (version 7.0.1), in accordance with the guidelines of the European and American Societies of Echocardiography. All echocardiographic parameters were measured in series, and the final value for each parameter was obtained as the average of repeated measurements across consecutive cardiac cycles. For each participant, values from the two examinations were averaged and used for analysis.

Statistical analysis

The results were processed using standard statistical methods in SPSS Statistics version 21.0 (IBM Corp., Armonk, NY). Continuous variables are presented as mean ± standard deviation (SD) if approximately normally distributed and as median (interquartile range (IQR) if non-normally distributed (normality was assessed using Shapiro-Wilk and/or visual inspection). Both descriptive and analytical statistical methods were applied. Linear and multiple regression analyses were used to assess associations between BP measures and LVMI. Associations were examined using LVMI as a continuous outcome, while Receiver operating characteristic (ROC) curve analysis evaluated discrimination of LVH (yes/no). Multiple regression models were constructed to adjust for potential confounding variables, including age, anemia, and two-month average volume overload. ROC curve analysis was performed to evaluate the ability of the average two-month pre-dialysis SBP to discriminate between the presence and absence of LVH and to identify an optimal cutoff value. The optimal cutoff was selected using the Youden index (sensitivity + specificity − 1). Sensitivity, specificity, and area under the curve (AUC) were calculated. Based on the ROC-derived cutoff, patients were categorized by SBP, and an unadjusted odds ratio (OR) with a 95% confidence interval (CI) was calculated from a 2×2 contingency table to estimate the odds of LVH. A p-value of less than 0.05 was considered statistically significant.

Ethical consideration

All participants provided written informed consent for the use of their medical data. The study was conducted in accordance with the Declaration of Helsinki and the principles of good scientific practice. Additionally, all participants had previously provided written informed consent for HD treatment and diagnostic procedures. The research protocol was approved by the Ethics Committee of the Faculty of Medicine, University of Banja Luka (18/4.22/17).

## Results

Patient characteristics

Baseline clinical and laboratory characteristics of the HD patients are summarized in Table 1. The median duration of dialysis treatment was 3.9 years (range: 0.5-6.2 years). Ischemic heart disease was present in 24 patients (25%). The median hemoglobin level during the month when echocardiography was performed was 115 g/L (IQR: 108-120), while the average two-month hemoglobin level was 114.5 g/L (IQR: 108-120). The two-month average volume overload was 11 ± 6%. The median LVMI was 117.5 g/m² (IQR: 98.7-142.9); in men, it was 122.9 g/m² (IQR: 100-148), and in women, 112.2 g/m² (IQR: 96.6-130.9). LVH was observed in 44 patients (45%).

**Table 1:** Clinical and echocardiographic characteristics of the study population IQR: interquartile range; SD: standard deviation; LVMI: left ventricular mass index

Variable	Value
Number of patients	97
Ischemic heart disease, n (%)	24 (25%)
Left ventricular hypertrophy, n (%)	44 (45%)
Duration of dialysis treatment, years, median (min–max)	3.9 (0.5–6.2)
Hemoglobin, g/L, median (IQR)	115 (108–120)
Two-month average hemoglobin, g/L, median (IQR)	114.5 (108–120)
Two-month average volume overload, %, mean ± SD	11 ± 6
Overall LVMI, g/m^2^, median (IQR)	117.5 (98.7-142.9)
LVMI in males, g/m², median (IQR)	122.9 (100.1–148.2)
LVMI in females, g/m², median (IQR)	112.2 (96.6–130.9)

Blood pressure measurements

Pre-dialysis arterial BP measurements are presented in Table 2. The single (mid-week) pre-dialysis SBP was 151.00 mmHg (IQR: 140.25-157.50), and the DBP was 75.00 mmHg (IQR: 68.00-82.75). The average two-month SBP was 153.57 mmHg (IQR: 140.46-164.96), and the average DBP was 76.72 mmHg (IQR: 68.58-83.16).

**Table 2:** Pre-dialysis single and average two-month arterial blood pressures For each patient, the two-month average BP was calculated as the mean of all pre-dialysis measurements over two months; values in the table are presented as median (IQR) across patients IQR: interquartile range

Pressures	Single		Average (two-month)	
-	Systolic, mmHg	Diastolic, mmHg	Systolic, mmHg	Diastolic, mmHg
Median	151.00	75.00	153.57	76.72
25th–75th percentile (P25–P75)	140.25–157.50	68.00–82.75	140.46–164.96	68.58–83.16

Linear regression analyses

**Figure 1:**
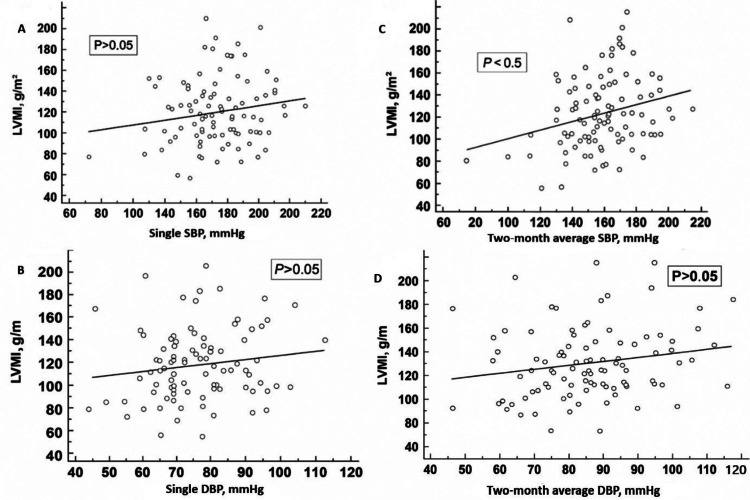
Scatter diagrams with linear regression lines (A) single SBP and LVMI; (B) single DBP and LVMI; (C) two-month average SBP and LVMI; (D) two-month average DBP and LVMI SBP: systolic blood pressure; DBP: diastolic blood pressure; LVMI: left ventricular mass index

Linear regression between single pre-dialysis SBP values and LVMI (Figure 1A) did not show a statistically significant association (p > 0.05). No statistically significant associations were observed between single DBP values and LVMI (Figure 1B), nor between average two-month DBP values and LVMI (Figure 1D) (p > 0.05 for both). However, a significant positive association was found between average two-month pre-dialysis SBP values and LVMI (Figure 1C; p < 0.05).

Multiple regression analyses

Multiple linear regression analyses were performed to assess the association between BP measures and LVMI after adjustment for age, hemoglobin, and volume overload. Single pre-dialysis SBP was not significantly associated with LVMI (β = 0.1339, p = 0.4197). In contrast, average two-month pre-dialysis SBP was significantly associated with LVMI (β = 0.3847, p = 0.0433). Single pre-dialysis DBP was also not significantly associated with LVMI (β = 0.4284, p = 0.1852). In the model including the average two-month pre-dialysis DBP, DBP was not significantly associated with LVMI (β = 0.5621, p = 0.0656).

Receiver operating characteristic (ROC) analysis

**Figure 2:**
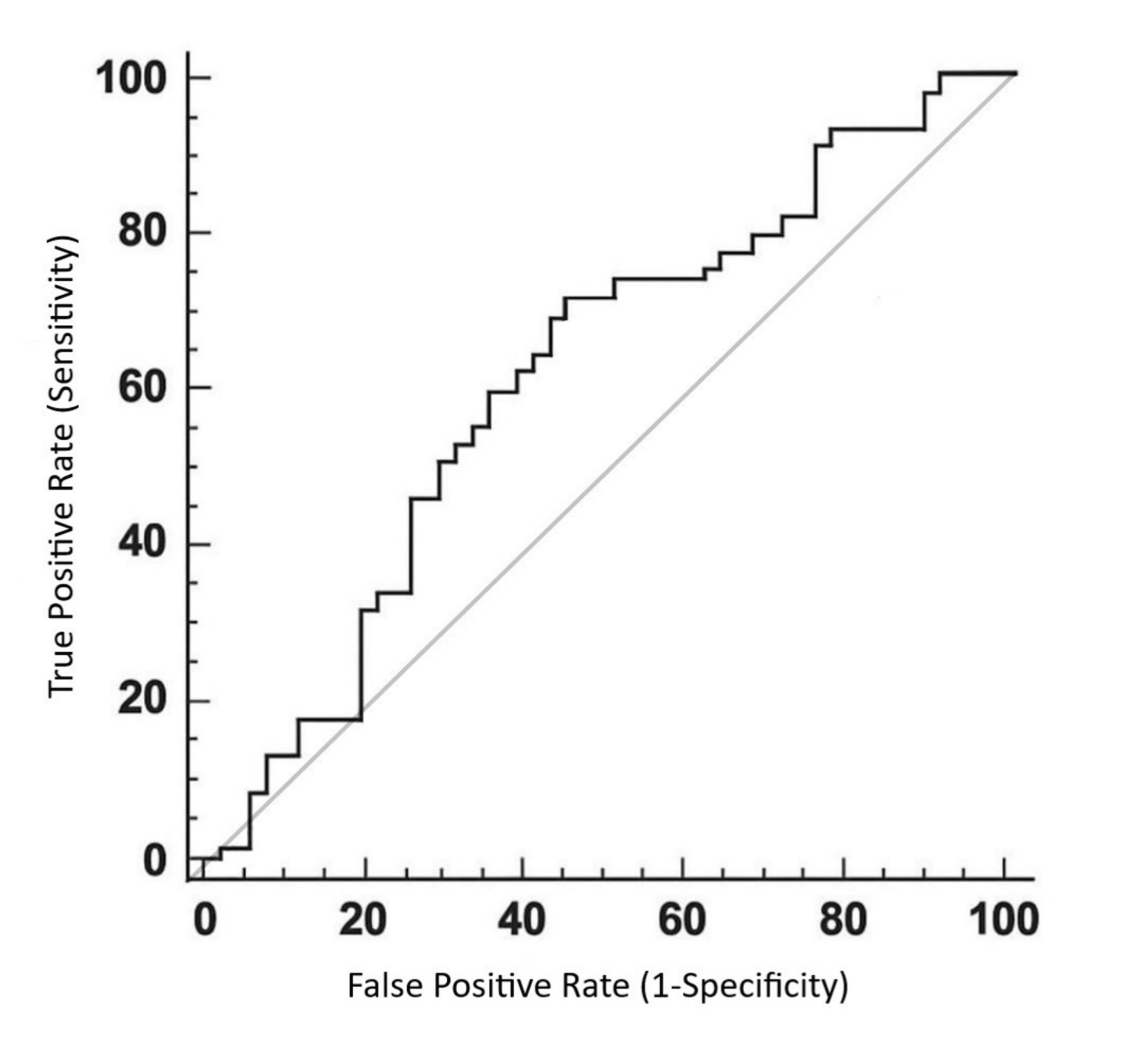
ROC curve for discrimination of prevalent left ventricular hypertrophy using average two-month pre-dialysis systolic blood pressure The curve illustrates the diagnostic performance at the optimal cutoff value (> 150 mmHg), with an AUC of 0.619 ROC: receiver operating characteristic; AUC: area under the curve

ROC analysis was performed as an exploratory assessment of the ability of the average two-month pre-dialysis systolic BP to discriminate prevalent LVH within the study cohort. The identified cutoff value was > 150 mmHg, with a sensitivity of 70.45% and a specificity of 55.77%. The AUC was 0.619 (95% CI: 0.515-0.717; p = 0.039), indicating modest discriminatory ability. Using this ROC-derived cutoff, patients with an average two-month pre-dialysis SBP > 150 mmHg had higher unadjusted odds of LVH compared with those with SBP ≤ 150 mmHg (OR = 3.11, 95% CI: 1.34-7.24; p = 0.039) (Figure 2).

## Discussion

The primary aim of this study was to examine whether the average two-month pre-dialysis BP values are associated with target organ damage, specifically LVH, in patients undergoing chronic HD. Our results demonstrated that the average two-month pre-dialysis SBP was associated with increased LVMI. In contrast, single SBP measurements and both average and single DBP values were not significantly associated with LVMI. Although the two-month average DBP showed a borderline association with LVMI (p = 0.066), it was not statistically significant. Volume overload and lower hemoglobin were independently associated with higher LVMI, supporting the role of volume status and anemia in LV remodeling. Although a threshold of > 150 mmHg emerged from the ROC analysis, its discriminatory performance was modest, and it should be considered exploratory rather than clinically definitive. Since this cutoff was derived and tested in the same cohort, its performance may be overestimated. This finding reinforces the clinical relevance of the ROC-derived cutoff and supports the concept that chronic systolic pressure load, rather than isolated BP measurements, plays a key role in left ventricular remodeling in HD patients. The observed increase in odds aligns with previous reports linking sustained pre-dialysis systolic hypertension to adverse cardiac structural changes and further emphasizes the importance of long-term BP control in this population.

These findings are broadly consistent with previous studies reporting an association between higher pre-dialysis SBP and increased LVMI or LVH in HD patients [12-16]. However, the available evidence is methodologically heterogeneous. Some earlier studies included larger samples or longitudinal follow-up, allowing stronger inference regarding sustained BP burden and progressive left ventricular remodeling [13,15,16], whereas others differed in patient population, adjustment strategy, and BP measurement approach [12,14]. In comparison with these reports, the association observed in our cohort was more modest and should be interpreted cautiously, particularly given the single-center setting, relatively small sample size, and the absence of longitudinal follow-up for incident or progressive LVH. Accordingly, our findings are directionally consistent with the existing literature but weaker in evidentiary strength than larger or longitudinal studies [12-16].

This interpretation is also relevant in the broader context of BP management in HD patients. Current guidance acknowledges the importance of pre-dialysis SBP assessment, but optimal BP targets in this population remain controversial because of measurement limitations, volume-related influences, and the complex relationship between BP and outcomes [17-21]. In addition, pre- and post-dialysis BP measurements are variable and less reproducible than out-of-center measurements, while ambulatory and home BP monitoring have shown stronger associations with LVH and cardiovascular outcomes [22-25]. Our findings, therefore, do not establish the superiority of pre-dialysis BP as a measurement modality. Rather, they suggest that, within the practical limitations of routine in-center assessment, a two-month average pre-dialysis SBP may better reflect sustained hemodynamic burden than a single-session value. The observed threshold of approximately 150 mmHg should be interpreted cautiously as an exploratory, cohort-specific finding rather than a clinically validated cutoff [17,22-25].

This study has several limitations. Due to the observational study design, causal relationships between BP measurements and LVH cannot be established. First, it was not double-blinded and lacked a control group, which limits causal inference and may introduce bias. Second, as an observational cohort study conducted at a single center with a relatively small sample size (n = 97), the generalizability of the findings is limited. Third, the study did not assess patient adherence to antihypertensive therapy or to home or ambulatory BP monitoring, which are known to influence outcomes in dialysis populations. A further limitation of this study is the lack of systematically available data on antihypertensive treatment regimens and treatment adherence. These factors may have influenced pre-dialysis BP values and left ventricular remodeling and therefore could not be accounted for in the present analysis.

Despite these limitations, the findings of this study provide valuable insights into the pathophysiology and epidemiology of LVH in HD patients. They help identify potentially modifiable risk factors, which could, in turn, lower the risk of LVH and improve patient survival through more effective BP management and cardiovascular risk reduction strategies.

## Conclusions

The findings of this study suggest that a higher two-month average pre-dialysis SBP was modestly associated with greater left ventricular mass in patients undergoing chronic HD, whereas single pre-dialysis BP measurements were not. The observed threshold of approximately 150 mmHg should be interpreted cautiously as a finding from the present analysis rather than as a clinically validated cutoff. Larger prospective studies are needed to confirm these findings and better define the clinical relevance of longitudinal pre-dialysis BP assessment.
